# Job performance in healthcare: a systematic review

**DOI:** 10.1186/s12913-021-07357-5

**Published:** 2022-02-04

**Authors:** Marcel Krijgsheld, Lars G. Tummers, Floortje E. Scheepers

**Affiliations:** 1 School of Governance, Utrecht University, Utrecht, The Netherlands; 2 Department of Psychiatry, Brain Center, UMC, Utrecht, The Netherlands

**Keywords:** Systematic review, Job performance, Task performance, Contextual performance, Adaptive performance, Counterproductive work behaviour, Healthcare

## Abstract

**Background:**

Healthcare organisations face major challenges to keep healthcare accessible and affordable. This requires them to transform and improve their performance. To do so, organisations must influence employee job performance. Therefore, it is necessary to know what the key dimensions of job performance in healthcare are and how these dimensions can be improved. This study has three aims. The first aim is to determine what key dimensions of job performance are discussed in the healthcare literature. The second aim is to determine to which professionals and healthcare organisations these dimensions of job performance pertain. The third aim is to identify factors that organisations can use to affect the dimensions of job performance in healthcare.

**Methods:**

A systematic review was conducted using the Preferred Reporting Items for Systematic Reviews and Meta-Analyses (PRISMA) statement. The authors searched Scopus, Web of Science, PubMed, and Google Books, which resulted in the identification of 763 records. After screening 92 articles were included.

**Results:**

The dimensions – task, contextual, and adaptative performance and counterproductive work behaviour – are reflected in the literature on job performance in healthcare. Adaptive performance and counterproductive work behaviour appear to be under-researched. The studies were conducted in different healthcare organisations and pertain to a variety of healthcare professionals. Organisations can affect job performance on the macro-, meso-, and micro-level to achieve transformation and improvement.

**Conclusion:**

Based on more than 90 studies published in over 70 journals, the authors conclude that job performance in healthcare can be conceptualised into four dimensions: task, contextual and adaptive performance, and counterproductive work behaviour. Generally, these dimensions correspond with the dimensions discussed in the job performance literature. This implies that these dimensions can be used for further research into job performance in healthcare. Many healthcare studies on job performance focus on two dimensions: task and contextual performance. However, adaptive performance, which is of great importance in constantly changing environments, is under-researched and should be examined further in future research. This also applies to counterproductive work behaviour. To improve job performance, interventions are required on the macro-, meso-, and micro-levels, which relate to governance, leadership, and individual skills and characteristics.

## Background

Together with governments and policymakers, healthcare organisations face major challenges to ensure healthcare remains accessible and affordable. This requires healthcare organisations to transform and improve their performance. These challenges cannot be met without the involvement and excellent performance of healthcare employees.

The Organisation for Economic Cooperation and Development (OECD) expects that in 2050, almost 27% of the population will be over 65 years old and more than 10% will be over 80 [1]. This may lead to increasing demand for healthcare. According to the OECD, healthcare expenditure in terms of gross domestic product will grow from 8.8% in 2017 to 10.2% in 2030 in OECD countries [1]. A record amount of money is being spent on healthcare, and this is expected to further increase due to pressure arising from, among other factors, an ageing population. However, advances in medical technology and rising public expectations regarding healthcare services also contribute to increasing health expenditure [2, 3]. Accessibility is not the only challenge arising from an ageing population and the consequent increasing demand for care; a shortage of healthcare professionals is another major challenge healthcare organisations face [4, 5]. All these challenges make healthcare perhaps one of the most important areas in which the change and improvement of organisational performance are necessary [2]. As healthcare is mainly people work, change and improvement in organisational performance will be closely linked to the performance (i.e., the actions and behaviours) of employees [6]. In other words, the job performance of healthcare professionals is of crucial importance to achieve organisational goals [6–8].

Job performance has been widely discussed and conceptualised in various ways [8]. This is reflected in Koopmans et al.’s [9] systematic review, in which the authors identify 17 generic and 18 job-specific frameworks. The job-specific frameworks in that study relate to the army and employees and management in the service and sales sector. However, Greenslade and Jimmieson’s (2007) framework was developed for the healthcare sector [10] based on Borman and Motowidlo’s theoretical model [11]. Based on the 35 frameworks Koopmans et al. identify four main dimensions: task performance, contextual performance, adaptive performance, and counterproductive work behaviour [9].

Task performance has a direct relationship with the organisational technical core [11–14]. The term refers to direct activities (such as treating patients) and indirect activities (such as hiring nurses) that are a formal part of a worker’s job [15]. Task performance is seen as an encompassing dimension that also includes aspects such as task behaviour [16], job and non-job specific tasks [17], role performance [18], technical activities [19], and action orientation [20]. Contextual performance includes, among other items, interpersonal behaviour [16], organisational citizenship behaviour [21], extra role performance [22], and peer team interaction [23]. Contextual performance concerns the broader organisational, social, and psychological environment in which a technical core must function [11–14]; it includes activities such as volunteering for extra work and maintaining good interpersonal relationships [15]. Adaptive performance refers to the extent to which an individual adapts to changes in work systems or work roles [9]. It is also defined as adaptability and pro-activity [24] and creative performance [21]. Attention towards adaptive performance has increased in recent decades due to the dynamic nature of work environments [25]. In earlier frameworks, adaptive performance was seen as a separate dimension [26–28] instead of a component of contextual performance [29]. Finally, counterproductive work behaviour refers to behaviour that is harmful to the performance of an organisation [30]. It includes, for instance, off-task behaviour, unruliness, theft, drug abuse [29], absenteeism (not attending work) and presenteeism (attending work while ill [31–33];).

To change and improve the performance of healthcare professionals, and thus the performance of healthcare organisations, it is important to determine whether the four dimensions can be used as a reference for job performance research in healthcare. Although Greenslade and Jimmieson (2007) propose a framework, it focuses specifically on nurses and only includes the task and contextual performance dimensions, thus having little applicability in healthcare research in general. Therefore, it is important to determine how job performance in healthcare is treated in the research literature and whether it relates to the dimensions of task, contextual, and adaptive performance and counterproductive work behaviour. To arrive at findings about whether the four dimensions can be applied to the broad field of healthcare, it is important to investigate in which sectors of healthcare and in relation to which professionals the dimensions have been used in research. Finally, to change and improve the performance of the healthcare professional, it is relevant to determine how and at which level organisations can implement changes to affect job performance. In summary, the purpose of this review is to answer the following questions:

1. *Which of the four job performance dimensions are described in studies focusing on job performance in healthcare?*
2. *To which professionals and health organisations do the dimensions of job performance discussed in the studies pertain?*
3. *How and on which level can organisations affect the job performance of healthcare professionals?*

This research was accomplished by conducting a systematic literature review. The method section describes the process of identification, screening, and assessing the eligibility of studies. The results section begins with an overview that sets out the distribution of the studies. The overview reveals in which year, and in which journal the articles were published. It also details whether studies were carried out in developed or developing countries. Further, this paper explains how it assesses the methodological quality of the studies. Following this overview, this paper presents the answers to the research questions, beginning first with the job dimensions identified in the selected studies, and then proceeding to an analysis of the type of organisations the studies examined and the healthcare professionals to which the studies pertain. Finally, the results section describes the factors that can affect job performance at different organisational levels. The discussion section discusses the results and reflects on a few of this paper’s limitations. The conclusion section provides suggestions that can be used for future research on job performance in healthcare based on this study’s findings.

## Methods

The literature search was conducted using the Preferred Reporting Items for Systematic Reviews and Meta-Analyses (PRISMA) statement [34]. To find eligible studies, four databases were searched: Scopus, Web of Science, PubMed, and Google Books. The goal of the research strategy was to find articles and books that relate to job performance in healthcare and include a broad scope of healthcare professionals. The search strategy is detailed in Appendix A.

### Eligibility criteria

Studies included in the review must meet the following criteria. They must relate to job performance in the field of healthcare. Job performance or comparable terms, such as work performance or work behaviour, must appear in the title or abstract. Studies that examine at least one of the four dimensions or related terms are also eligible. Studies published between 1996 and December 2019 were selected. As part of the pragmatic approach to gathering literature, only studies written in English were considered. All articles published in international journals that were selected for this study must have been peer-reviewed.

### Study selection

Through the search strategy, 763 records were identified, including four books. After 17 duplicates were removed, the titles and abstracts of the remaining 747 records were screened. This resulted in the exclusion of 497 records (including three books). Although the studies are related to healthcare, job performance was not the main objective of these studies. For example, a few studies examine musculoskeletal disorders and their impact on nursing tasks [16, 17]. Other studies focus on job satisfaction [18, 19]. After the exclusion of these 497 studies, the authors read the remaining 250 articles in detail and analysed their eligibility. This resulted in the exclusion of another 158 studies. The grounds for exclusion are as follows. Studies that focus on a specific task, such as working with electronic healthcare systems [20, 21], radiation therapy [35], cervical screening [36], and communication in the operating theatre [24, 25], were excluded.

**Fig. 1:**
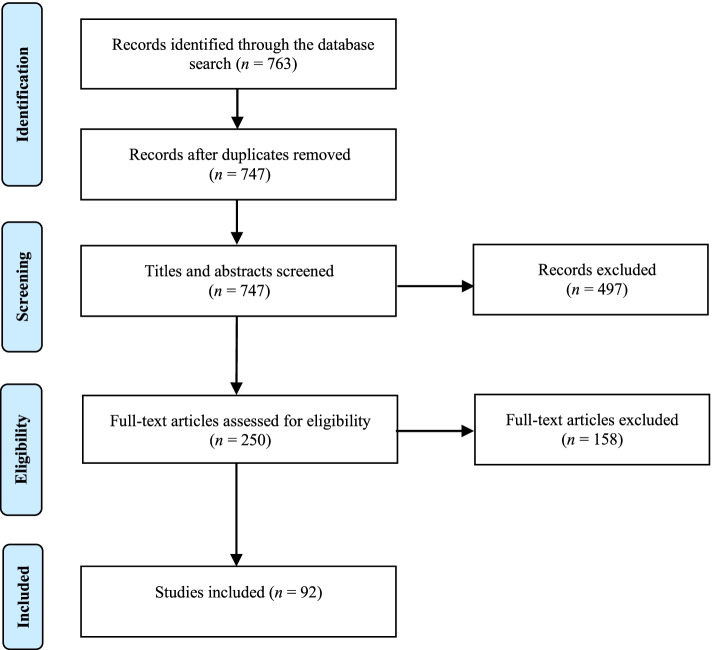
Flowchart study selection

Full-text articles were not available for two studies. After completing the process of screening and analysing the articles, a total of 92 articles, including one book chapter, met the eligibility criteria. The study selection process is depicted schematically in Fig. 1 using the PRISMA flowchart [34].

After categorising the articles by year of publication and the journals and countries in which they were published, the methodological quality of the studies was assessed using the integrated quality criteria for the review of multiple study designs [37]. Studies that could not be assessed using the ICROMS tool were assessed using the Standard Quality Assessment Criteria for Evaluating Primary Research Papers [38]. Because not all the selected studies directly refer to task, contextual, or adaptive performance or counterproductive work behaviour, it was imperative to assign terms, such as nursing work, tasks, or activities and indirect or direct care [27, 28] to one of the dimensions. The assignment of the terms was accomplished using the definitions of the four dimensions. To determine whether the dimensions of job performance were used in the broad field of health care, the type of organisation in which job performance was studied was examined. In addition, it was analysed to which professionals these studies related. Finally, the factors influencing job performance were categorised into macro-, meso-, and micro-level factors. All coding can be viewed on the Open Science Framework (OSF) database.

## Results

Before answering the research questions, this paper provides an overview that sets out the distribution of the studies. The overview reveals in which year and in which journal the articles were published. It also shows whether the studies were carried out in developed or developing countries. Results of the assessment of the methodological quality of the studies are provided below.

### Distribution of the studies

Table 1 reveals that most studies (82.6%) were conducted in developed countries (e.g., [39–41]), with the United States being the most common study location (29.4% of all studies; e.g., [42–44]). With regard to developing countries, China was the most common study location (e.g., [45, 46]).

**Table 1:** Distribution of the articles in developed and developing countries

***Developed countries*** ^***a***^	***References*** ^***b***^	***N (%)***	***Developing countries*** ^***a***^	***References*** ^***b***^	***N (%)***
United States	[4, 11, 18, 23–25, 27, 29, 33, 40, 41, 49, 56, 58, 61, 62, 66, **68**^**c**^, 71, 75, 78, 81–83, 85, 87]	27 (29.4)	China	[5, 7, 19, 28]	4 (4.3)
Australia	[9, 26, 30, 31, 32, 35, 37, 39, **88**]	9 (9.8)	Korea	[13, 20]	2 (2.2)
Germany	[14, 21, 22, 55, 59, 77, 80]	7 (7.6)	Nigeria	[10, 12]	2 (2.2)
United Kingdom	[16, 36, 45, 54, 74, 86, **88**, 92]	7 (7.6)	Malaysia	[8, 91]	2 (2.2)
Canada	[15, 34, 46, 51, 53, 65]	6 (6.5)	Saudi Arabia	[1, 3]	2 (2.2)
Netherlands	[6, 44, 67, **68**]	4 (4.3)	India	[2]	1 (1.1)
Belgium	[38, 72, 84]	3 (3.2)	Iran	[17]	1 (1.1)
Israel	[48, 50, 63]	3 (3.2)	Pakistan	[69]	1 (1.1)
Italy	[52, 64, 73]	3 (3.2)	Turkey	[60]	1 (1.1)
Finland	[76]	1 (1.1)			
Japan	[44]	1 (1.1)			
New Zealand	[42]	1 (1.1)			
Norway	[79]	1 (1.1)			
Spain	[90]	1 (1.1)			
Sweden	[47]	1 (1.1)			
Taiwan	[89]	1 (1.1)			
**Total**		**76 (82.6)**			**16 (17.4)**

^a^ Based on the IMF World Economic Outlook Database, October 2018: https://www.imf.org/en/Publications/WEO/weo-database/2018/October

^b^ See Appendix B. ^c^ Studies conducted in two countries

The articles included in this review were published in 76 different journals (Appendix C). The journals can be divided into healthcare fields, such as nursing [47], medicine [42], healthcare [48], and psychology [49], and into journals with a focus on specific topics, such as maternity [50]*,* ergonomics [51], and critical care [52]. Almost 20% of the articles were published in the following four journals: *BMC Health Services Research,* the *Journal of Advanced Nursing*, the *International Journal of Medical Informatics,* and the *Journal of Managerial Psychology*. Most of the studies were conducted in a single country, which raises questions about their external validity.

**Fig. 2:**
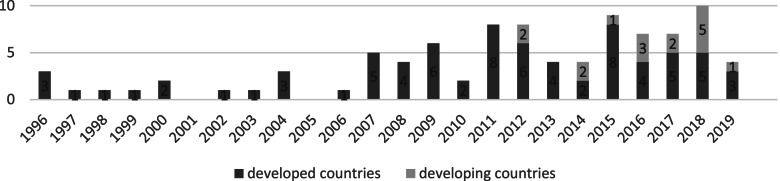
Number of publications on job performance in healthcare, 1996–2019

Figure 2 illustrates the publication years of the studies, divided into publications in developed and developing countries. It indicates that job performance in healthcare has been studied almost continuously over the years and is still of interest. Figure 2 also suggests that the interest in job performance in healthcare has increased in developing countries over the last decade.

### Design and quality of the studies

To assess the methodological quality of the studies, the ICROMS quality assessment tool was used [37]. The tool provides a comprehensive set of general and specific quality criteria for randomised controlled trials (RCTs), controlled before-after (CBA) studies, non-controlled interrupted time series (NCITS) studies, cluster-randomised controlled trials (cRCTs), and non-controlled before-after (NCBA) studies. The ICROMS tool also provides a clear and transparent scoring system with a minimum required score per study design. The results of the study designs are listed in Table 2. The ICROMS scores of the assessed studies are shown in the OSF database. Qualitative and cohort studies, CBA studies, RCTs, and NCITS studies all achieved the minimum required score. Although the minimum required score was achieved in these studies, room for improvement exists. About 60% of the studies suffer from selective outcome reporting due to unavailable study protocols. Clear statements as to whether or not the studies were selectively reported did not solve the issue with the lack of protocols. On average, only the NCBA studies failed to meet the minimum required score because no baseline measurements were conducted, and no attempt was made to mitigate the effect of not having a control group. Although the quality of these NCBA studies is low, one can nonetheless provide some commentary on them. For instance, not all ICROMS items could be evaluated because it is unclear whether the criteria were met. The lack of evidence that this cannot be ascertained from an article does not mean that the criteria have not been applied. Researchers can accomplish improvement by providing a better description of the method of subject selection and its characteristics.

**Table 2:** Results of the assessment of the methodological quality of the studies, assessed using ICROMS

Design category	# of studies (%)	Mean score (range); max possible score	Minimum required score
Qualitative	37 (80.5)	21.6 (17–26); 26	16
Cohort	3 (6.5)	23.7 (23–25); 26	18
Controlled before-after (CBA)	2 (4.4)	27.5 (27–28); 28	18
Non-controlled-before-after (NCBA)	2 (4.4)	19.5 (19–20); 30	22
Non-controlled interrupted time series (NCITS)	1 (2.1)	25.0 (25); 28	22
Randomised controlled trial (RCT)	1 (2.1)	25.0 (25); 32	22
Total	46 (100)		

The ICROMS tool has a scope for further development of quality criteria applicable to additional study designs, such as surveys and cross-sectional studies [37]. Therefore, studies that rely solely on data from questionnaires could not be assessed using the ICROMS tool. These studies (e.g., [30, 53]) were assessed using the Standard Quality Assessment Criteria for Evaluating Primary Research Papers [38]. The overall score ranged from 0.72–1.0 (mean: 0.91, standard deviation: 0.07).

### Dimensions of job performance

The first research question examines which of the four dimensions of work performance (i.e., task, context, and adaptive performance and counterproductive work behaviour) are described in studies of work performance in healthcare. The results show that these dimensions are applicable to work performance in healthcare.

The review of the literature revealed studies that directly refer to Motowidlo et al. [11], who classify and define job performance as task and contextual performance (e.g., [46, 49, 54]). Studies were also found that directly refer to Greenslade and Jamieson [10], who suggest a model based on Motowidlo and Van Scotter’s [55] classification of methods to measure the job performance of nurses, which is directly linked to two dimensions, task and contextual performance (e.g., [56–58]). Studies referring to organisational citizen behaviour (e.g., [59, 60]) were classified as contextual performance because there is significant overlap between the definitions of organisational citizen behaviour and contextual performance [9]. Overlap was also found in studies that directly refer to counterproductive work behaviour (e.g., [61, 62]). In addition to the studies that directly refer to the dimensions of job performance, other studies described task, skill, and behavioural performance without a direct reference to the dimensions of job performance. The definitions [9] listed in Table 3 were used by the researchers to assign these tasks, skills, and behaviours to one of the dimensions of job performance if they were in alignment with those definitions.

**Table 3:** Definitions of the four dimensions of job performance based on Koopmans et al.’s review (2011)

Task performance	Has a direct relationship to an organisational technical core and refers to direct or indirect activities that are formally part of a worker’s job
Contextual performance	Maintains the broader organisational, social, and psychological environments in which a technical core must function
Adaptive performance	The extent to which an individual adapts to changes in work systems or work roles
Counterproductive work behaviour	Behaviour that is harmful to the well-being of an organisation

Patient feeding [63], direct patient contact [64], scheduling toileting [65], and speaking with other professionals concerning patient care [66] are examples of tasks that were attributed to the task performance dimension because these examples are part of a healthcare professional’s job. Visiting unit and hospital meetings [67], continuing professional development [68], and tutoring trainees [69] were attributed to contextual performance because these examples contribute to the improvement of an organisation overall. The willingness to implement organisational changes [70] and the eagerness to require professional information [71] are examples of behaviours that were attributed to adaptive performance because they are important to adapt to changes in work systems and roles. Purposely failing to help a colleague [72] and rude behaviour among supervisors [73] are examples of behaviours that were attributed to the dimension of counterproductive work behaviour because these behaviours can lead to employee illness and increase turnover and therefore harm an organisation’s well-being. A full description of the allocation of the studies within this paper’s sample to the dimensions is available on the OSF database. All tasks, skills, and behaviours can be assigned to one of the four dimensions of job performance. Along with the studies that directly refer to these dimensions, Table 4 lists the assignment results.

**Table 4:** The distribution *(or combinations)* of dimensions of job performance

***Dimensions***	***References*** ^***a***^	***N (%)***
Task performance	[***1***, ***3***, 4, ***5***, 6, ***8***, ***9***, ***11***, ***13***, 14, ***15***, 16, 17, ***19***, *20*, 21, ***22***, ***24–26***, 28, ***29–32***, 33, ***34–37***, 38, ***39***, 40, 41, ***42***, 44, ***45***, 46, 47, ***48–50***, 51, ***52***, ***54***, 55, ***57–62***, *63*, ***64***, ***67***, 68, 69, 70, 71, *74*, ***75***, 76, ***77***, ***78***, *79*, ***80***, ***81***, 82, 83, *84*, ***85–87***, 88, ***89***]^*b,c,d,e*^	73 (47.1)
Contextual performance	[***1***, 2, ***3***, ***5***, 6, ***8***, ***9***, ***11***, ***13***, ***15***, ***19***, *20*, ***22***, ***24–26***, ***29–32***, ***34–37***, ***39***, ***42***, ***45***, ***48–50***, ***52***, 53, ***54***, 55, *56*, ***57–62***, *63*, ***64***, 65, ***67***, 68, 69, *72*, 73, *74*, ***75***, ***77***, ***78***, ***80***, ***81***, *84*, ***85–87***, ***89***, 90]^b,c,d,e^	61 (39.3)
Counterproductive work behaviour	[7, 10, 12, 18, *20*, 23, 27, *56*, *63*, *72*, *74*, *84,* 91] ^*d,e*^	13 (8.4)
Adaptive performance	[6, 43, 55, *56*, 66, 68, 69, *79*]^*c,e*^	8 (5.2)
All four dimensions	No references	0 (0.0)
		**155 (100)**

^a^ See Appendix B. ^b^ References in bold italics concern studies in which task and contextual performance both occur. ^c^ Underlined references concern studies that bring together task, contextual, and adaptive performance. ^d^ References in italics refer to studies about task and contextual performance and counterproductive work behaviour. ^e^ Underlined and italicised references refer to studies with combined dimensions

The results reveal that over 47% of the studies focus on task performance, such as primary care tasks [36], supportive care [50], and manual tasks [74]. They also show a focus on contextual performance, which is about team interdependence, communication, synchronicity, coordination and confidence in interprofessional collaboration, and knowledge sharing [75]. A total of 45 studies investigates contextual performance in combination with task performance. This follows logically from Motowidlo et al.’s [11] frequently used definition of job performance. Thirteen studies focus on counterproductive work behaviour, which includes abuse, production deviance, sabotage, theft, absence, early and late arrival [61], workplace violence, verbal aggression, harassment, intimidation, threats, and bullying [76]. Only eight studies include the adaptive performance dimension; for example, some studies examine adopting electronic health record systems [77], adopting new innovations [71], creativity, or personal initiatives [59].

### Healthcare organisations and professionals

The second research question concerns the type of healthcare organisations in which the studies investigate job performance and the type of healthcare professionals to which the studies pertain. The studies examine job performance in several healthcare fields and with respect to various types of healthcare professionals. Table 5 lists the types of healthcare organisations the studies examine. It indicates that over 77% of the studies were performed in hospitals (e.g., [78, 79]), including in cardiology, general surgery, anaesthetics [80], and psychiatry [39] wards or in special hospitals such as children’s hospitals [45, 81]. Other studies investigate job performance in hospices [82], organisations for patients with special needs [59], and nursing homes [36]. In six studies, the research was performed in both hospitals and other healthcare organisations. One study did not specify the type of healthcare organisation the authors studied [83].

**Table 5:** Healthcare organisations where research into job performance was conducted

***Type of healthcare organisations***	***References*** ^***a***^	***N (%)***
Hospitals	[1–22, ***23***, 24, 27***–***35, 36, 37, 39, 42, ***43***, 46, 49, 51, 52, ***53***, ***54***, 58–66, ***67***, 68, 69, 70, 72, 78, 80–91]^b^	75 (77.3)
Nursing homes	[***23***, 26, 38, 41, 45, 46, 47, 55, ***67***]^c^	9 (9.3)
Community health centres	[25, 40, 48, ***53***, ***54***, 57, 71, 79]	8 (8.3)
Home care	[***23***, ***43***, 44]	3 (3.1)
Occupational health service	[50]	1 (1.0)
Not specified	[56]	1 (1.0)
		**97 (100)**

^a^ See Appendix B. ^b^ References in bold italics indicate studies conducted in both hospitals and other health care organisations. ^c^ Includes homes for special needs patients and hospices, outpatient care, pharmacies, and community centres

About 52% of studies in the sample concern the job performance of nurses (e.g., [53, 84]; see Table 6). Besides general nurses, several studies also focus on intensive care nurses [52, 85] and maternity nurses [50]. In about 26% of the studies, physicians (e.g., [42, 86]), such as paediatricians [81] and gynaecologists [77], are the focus of attention. Eighteen studies investigate the job performance of other healthcare professionals, such as pharmacists [87, 88], lab technicians [61], and administrative employees [72]. Five studies do not specify the type of professional the authors examined. Markon, Chiocchio, and Fleury discuss healthcare professionals in general [75].

**Table 6:** Investigated healthcare professionals in each study

***Disciplines***	***References*** ^***a***^	***N (%)***
Nurses	[1–5, ***6***, 8–11, *12*, 13, *15*, 17, 20, 23–25, ***27***, 28, 30, 31, *32*, 33, 35, 36, *38*, 39–42, 43–47, 49, 51, 52, 60, *61*, ***63****,* *64,* *65,* 67, ***68****,* 70, 73, *77*, ***82****,* 84, ***85***, 89, 90, 91]^b,c^	55 (52.4)
Physicians	[***6***, 7, 14, 16, 19, 21, 22, ***27****,* 29, 34, 37, 48, 50, ***63****,* 66, ***68****,* 69, 74, 76, 78, 79, 80, ***82****,* 83, ***85****,* 86, 87]^b^	27 (25.7)
Healthcare professionals ^d^	[*12*, *15*, 26, *32*, *38*, 53, 54, 56, 57, 58, 59, *61*, *64*, *65*, 71, 72, *77*, 88]^c^	18 (17.1)
Healthcare professionals (not specified)	[18, 55, 62, 75, 81]	5 (4.8)
		**105 (100)**

^a^ See Appendix B. ^b^ References in bold italics concern studies on both nurses and physicians. ^c^ References in italics that are underlined concern studies on nurses and other healthcare professionals. ^d^ Includes personal care workers, mental healthcare professionals, pharmacy staff, caregivers, administrative employees, final-year medical students, care assistants, administrative staff, counsellors, psychologists, pharmacists, social workers, lab technicians, and supervisors

### Factors affecting the job performance of healthcare professionals

To answer the third research question, which concerns factors that affect the healthcare professionals’ job performance, this study distinguishes between the macro-level (organisation), meso-level (management/team), and micro-level (individual). This distinction reveals that the job performance of healthcare professionals can be affected on all three levels.

On the macro-level, job performance can be affected by how an organisation is structured [82], the extent to which a healthcare professional perceives that they have organisational support [53, 73], and organisational culture [89]. Employee performance can flourish in an innovative atmosphere [71]. In contrast, job performance is likely to decrease in a toxic organisational climate and in cases where supervisors act abusively [61, 90]. Turnover of high-performing employees can also affect an organisation’s performance negatively [54].

At the meso-level, managerial support and supervision and training programmes contribute to job performance levels [75, 76, 91]. In addition, factors such as interdependence [75], team structure [88], and the presence of social support [57, 92] can affect job performance. Positive views towards work and innovation in organisations with employee-centred designs [93] contribute positively to job performance. Factors that negatively affect job performance on the meso-level include abusive supervision [94], limited resources, heavy workloads and dissatisfaction with co-workers [76], and burnout [95].

On the micro-level, the extent of work engagement, role clarity, and autonomy [53, 96], as well as employee skills and education levels [58], overwork [69], and the prevalence of multitasking [64] are relevant factors that influence job performance. Other relevant factors that influence job performance applies to employees’ personal characteristics, such as openness to change and extraversion [56, 67, 97], seeking challenges [70], eagerness [71], and creativity [59]. Low emotional intelligence [98] and Machiavellianism – pragmatic, emotionally detached, and task oriented as.

opposed to person oriented – affect job performance in a negative manner [45]. In summary, the governance of an organisation, the style of management or leadership, and the individual skills and characteristics of the professionals at an organisation can improve or diminish the performance of individual employees. This, in turn, can affect organisational performance (Table 7).

**Table 7:** Factors affecting job performance on the macro-, meso-, and micro-levels

Level	Factors that positively affect performance	Factors that negatively affect performance
Macro (organisation)	Organisational support Organisational structure Involved organisational culture	Toxic climate/culture Abusive supervision Turnover of high-performing employees
Meso (management/team)	Team structure Perceived interdependence Social supports	Abusive supervision Limited resources Heavy workloads Dissatisfaction with co-workers
Micro (individual)	Work engagement Role clarity Autonomy Skills and level of education Personal characteristics (openness to change, extraversion, eagerness, and creativity)	Low emotional intelligence Machiavellianism Burnout

## Discussion

To the best of the authors’ knowledge, this paper appears to be the first systematic review of the dimensions of job performance in healthcare, given that the study selection research process only produced one study that examine frameworks on job performance in healthcare. This one exception concerns Greenslade and Jimmieson’s framework; however, their study focuses specifically on nurses and thus is not broadly applicable to the field of healthcare [10]. The review in the instant paper also provides an important contribution by gathering knowledge on job performance in healthcare through an examination of articles published in 76 different journals. Most of these studies were conducted in single countries and often within the same types of healthcare organisations, which limits their generalisability. The interest in job performance in developing countries has only become apparent over the last decade. The methodological quality of the sample studies was assessed, revealing that most studies met the minimum required score. Although this minimum score was required, there is room for improvement in the literature, as over 60% of the studies suffer from selective outcome reporting due to the unavailability of study protocols. Along with improving generalisability, these issues should be considered in future research on this topic.

Studies concerning job performance in healthcare tend to apply at least one of the four dimensions of job performance. Studies without a direct reference to the task, contextual, or adaptive performance or counterproductive work behaviour dimensions offer descriptions of the activities, skills, and behaviours of healthcare employees. Based on the definitions of the dimensions, these activities, skills, and behaviours are attributable to at least one of the dimensions of job performance. Therefore, future studies about job performance in healthcare could be built on these dimensions.

Although the four dimensions do appear in healthcare literature concerning job performance, there is a discrepancy in the extent to which the dimensions have been studied. Task performance (49%) and contextual performance (39%) have been exhaustively investigated, whereas adaptive performance (8%) – which is also of great importance in constantly changing environments such as healthcare – appears to be under-researched. The same is true of the counterproductive work behaviour dimension, which can have a substantial and negative effect on job performance. Authors should consider this gap in job performance research in future research endeavours.

This review shows that scholars have studied the dimensions in different types of healthcare organisations and with reference to a variety of healthcare professionals. The main type of healthcare organisation the studies examine is hospitals and the departments and wards within them. About 22% of the studies were conducted in nursing homes, community centres, and home care organisations (among other organisations). Because most studies were conducted in hospitals, it was expected that most of the surveyed professionals would be physicians (26%) and nurses (52%). Other professionals the studies examine include mental healthcare professionals, psychologists, pharmacists, lab technicians, and supervisors. Consequently, the results show that the task, contextual, and adaptive performance and counterproductive work behaviour dimensions all apply to the broad field of healthcare and pertain to professions that exist within the healthcare sector. As such, these dimensions are useful for examining job performance in the broad context of healthcare and healthcare professionals.

This research not only investigated which dimensions of job performance can be used in the context of healthcare but also how and at what level these dimensions could be affected. The results show that the job performance of healthcare professionals can be affected on three levels. On the macro-level, the structure of an organisation, support for the board among an organisation’s employees, and organisational culture are examples of factors that affect job performance. At the meso-level, job performance can be affected to how management acts, how work is organised, and how teams function. On the micro-level, job performance is affected by employee motivation, the educational levels of the professionals in question, and employees’ personal characteristics. These levels are interdependent. Thus, organisations cannot simply improve the job performance of healthcare professionals in isolation from other efforts, and research aimed at improving job performance must be conducted with reference to these three levels. Given the apparently limited research regarding the adaptive performance and counterproductive work behaviour dimensions in healthcare, this paper suggests researchers investigate these dimensions with reference to the factors at the aforementioned levels to influence these dimensions.

### Limitations

The review set out in this paper has a few limitations. First, it is not certain that the review identified and covered all studies concerning job performance in healthcare. One reason for this is the fact that only English articles were eligible for inclusion based on the eligibility criteria. By including studies that were conducted in non-English speaking regions and in both developed and developing countries, this paper tries to reduce the impact of this potential limitation. Second, since the search criteria focused on at least one of the four dimensions, there is a possibility that other potential dimensions may not have emerged from the results. A possible third limitation is based on the fact that job performance is described in many ways, and there are many different terms that could be related to dimensions of job performance. Finally, the ratio between studies that were conducted in developed and developing countries within the sample implies a validation risk. However, studies that were conducted in either developed or developing countries are referred to in Greenslade and Jimmieson’s [10] and Motowidlo et al. [11] works. Despite these limitations, the findings in this review provide support for further research on job performance in healthcare.

## Conclusion

This research aimed to provide a concept that can be used for research on job performance in healthcare. Based on an examination of more than 90 studies published in over 70 journals, this research shows that job performance in healthcare can be conceptualised into four dimensions: task, contextual, and adaptive performance, and counterproductive work behaviour. While some of the studies directly refer to these dimensions, other studies describe tasks, skills, and behaviours without making direct reference to the four dimensions. However, these tasks, skills, and behaviours were assigned to one of the dimensions of job performance if they were in alignment with their definitions. In healthcare studies on job performance, the focus is on task and contextual performance. However, adaptive performance, which is of great importance in a constantly changing environment, is under-researched and should be considered a topic for future research. This is also suggested for the counterproductive work behaviour dimension. To improve job performance, interventions – in conjunction with one another – are required on the macro-, meso-, and micro-levels, which concern governance, leadership, and individual skills and characteristics.

## Data Availability

Data is available at https://osf.io/xn9r4/?view_only=aa9cf6c701644e1bac7bc30d853877be

## Appendix A

### Search strategies

**Scopus**: (TITLE ({job} OR {work} OR “worker*” OR {personnel} OR {staff} OR {professionals} OR {performance}) AND TITLE ({healthcare} OR {health-care} OR doctor* OR nurse* OR {nursing} OR hospital* OR physician*) AND TITLE-ABS-KEY ({task performance} OR {contextual performance} OR {adaptive performance} OR {counterproductive} OR {counter-productive}).

**Pubmed**: (“Work Performance”[Mesh] OR work performance[tiab] OR job performance[tiab]) AND (Task*[tiab] OR Contextual[tiab] OR Adaptive[tiab] OR Counterproductive[tiab] OR counter-productive[tiab]) AND (“Health Personnel”[Mesh] OR health personnel[tiab] OR healthcare personnel[tiab] OR care personnel[tiab] OR care worker*[tiab] OR healthcare provider*[tiab] OR care provider*[tiab] OR healthcare worker*[tiab] OR caregiver*[tiab] OR medical staff[tiab] OR hospital staff[tiab] OR hospital personnel[tiab] OR nurse[tiab] OR nurses[tiab] OR doctor*[tiab] OR physician*[tiab]).

**Web of Science**: TS = (job OR work OR worker* OR personnel OR staff OR professionals) AND TS = (“task performance” OR “contextual performance” OR “adaptive performance” OR “counterproductive behavio$r”) AND TS = (care OR healthcare OR doctor* OR nurse* OR hospital* OR physician*).

**Google Books**: “job|work performance” “Task|contextual|adaptive performance”|Counterproductive intitle:healthcare|care|doctors|nurses|hospital|physicians intitle:performance|teamwork|competency|job|work|potential|professional|skill|behavior|behaviour.

## Appendix B

### Articles referred to in Tables 1, 4, 5 and 6

1. Bhatti, M. A., Mat, N., & Juhari, A. S. (2018). Effects of job resources factors on nurse’s job performance (mediating role of work engagement). *International Journal of Health Care Quality Assurance*, *31*(8), 1000–1013.

2. Malik, N. (2018). Authentic leadership an antecedent for contextual performance of Indian nurses. *Personnel Review*, *47*(6), 1244–1260.

3. Bhatti, M. A., Alshagawi, M., & Syah Juhari, A. (2018). Mediating the role of work engagement between personal resources (self-efficacy, the big five model) and nurses’ job performance. *International Journal of Human Rights in Healthcare*, *11*(3), 176–191.

4. Schenk, E., Schleyer, R., Jones, C. R., Fincham, S., Daratha, K. B., & Monsen, K. A. (2018). Impact of Adoption of a Comprehensive Electronic Health Record on Nursing Work and Caring Efficacy. *CIN:Computers Informatics Nursing*, *36*(7), 331–338.

5. Tong, L. (2018). Relationship between meaningful work and job performance in nurses. *International Journal of Nursing Practice*, *24*(2).

6. Gordon, H. J., Demerouti, E., Le Blanc, P. M., Bakker, A. B., Bipp, T., & Verhagen, M. A. M. T. (2018). Individual job redesign: Job crafting interventions in healthcare. *Journal of Vocational Behavior*, *104*, 98–114.

7. Ying, L., & Cohen, A. (2018). Dark triad personalities and counterproductive work behaviors among physicians in China. *International Journal of Health Planning and Management*.

8. Zawawi, A. A., & Nasurdin, A. M. (2017). The impact of task characteristics on the performance of nursing teams. *International Journal of Nursing Sciences, 4*(3), 285–290.

9. Roche, M. A., Friedman, S., Duffield, C., Twigg, D. E., & Cook, R. (2017). A comparison of nursing tasks undertaken by regulated nurses and nursing support workers: a work sampling study. *Journal of Advanced Nursing*, *73*(6), 1421–1432.

10. Ugwu, L. I., Enwereuzor, I. K., Fimber, U. S., & Ugwu, D. I. (2017). Nurses’ burnout and counterproductive work behavior in a Nigerian sample: The moderating role of emotional intelligence. *International Journal of Africa Nursing Sciences*, *7*, 106–113.

11. Higgins, L. W., Shovel, J. A., Bilderback, A. L., Lorenz, H. L., Martin, S. C., Rogers, D. J., & Minnier, T. E. (2017). Hospital nurses’ work activity in a technology-rich environment: A triangulated quality improvement assessment. *Journal of Nursing Care Quality*, *32*(3), 208–217.

12. Gabriel, J. M. O. (2016). Supervisors’ toxicity as predictor of subordinates’ counter-productive work behavior in Nigerian public hospitals. *Journal of Applied Business Research*, *32*(5), 1363–1374.

13. Park, I. S., Suh, Y. O., Park, H. S., Ahn, S. Y., Kang, S. Y., & Ko, I. S. (2016). The job analysis of Korean nurses as a strategy to improve the Korean Nursing Licensing Examination. *Journal of Educational Evaluation for Health Professions*, *13*.

14. Wolff, J., McCrone, P., Patel, A., Auber, G., & Reinhard, T. (2015). A time study of physicians’ work in a german university eye hospital to estimate unit costs. *PLoS ONE*, *10*(3).

15. White, D. E., Jackson, K., Besner, J., & Norris, J. M. (2015). The examination of nursing work through a role accountability framework. *Journal of Nursing Management*, *23*(5), 604–612.

16. Brown, M., Shaw, D., Sharples, S., Jeune, I. L., & Blakey, J. (2015). A survey-based cross-sectional study of doctors’ expectations and experiences of non-technical skills for out of hours work. *BMJ Open*, *5*(2).

17. Heydari, A., Mazloom, R., Najar, A. V, & Bakhshi, M. (2015). Awareness and performance of Iranian nurses with regard to health economics: A cross-sectional study. *North American Journal of Medical Sciences*, *7*(9), 384–389.

18. Hamblin, L. E., Essenmacher, L., Upfal, M. J., Russell, J., Luborsky, M., Ager, J., & Arnetz, J. E. (2015). Catalysts of worker-to-worker violence and incivility in hospitals. *Journal of Clinical Nursing*, *24*(17–18), 2458–2467.

19. Yuan, Y., & Zhong, S. (2014). The compensation plan on doctors considering the contextual performance. *Advances in Intelligent Systems and Computing*. Business School, Sichuan University, Chengdu, 610,065, China.

20. Yun, S., Kang, J., Lee, Y.-O., & Yi, Y. (2014). Work Environment and workplace bullying among Korean intensive care unit nurses. *Asian Nursing Research*, *8*(3), 219–225.

21. Weigl, M., Hoffmann, F., Mueller, A., Barth, N., & Angerer, P. (2014). Hospital paediatricians’ workflow interruptions, performance, and care quality: a unit-based controlled intervention. *Europena Journal of Pediatrics*, *173*(5), 637–645.

22. Weigl, M., Mueller, A., Sevdalis, N., & Angerer, P. (2013). Relationships of Multitasking, Physicians’ Strain, and Performance: An Observational Study in Ward Physicians. *Journal of Patient Safety*, *9*(1), 18–23.

23. Estes, B. C. (2013). Abusive Supervision and Nursing Performance. *Nursing Forum*, *48*(1), 3–16.

24. Douglas, S., Cartmill, R., Brown, R., Hoonakker, P., Slagle, J., Schultz Van Roy, K., … Carayon, P. (2013). The work of adult and pediatric intensive care unit nurses. *Nursing Research*, *62*(1), 50–58.

25. Anderson, D. R., St. Hilaire, D., & Flinter, M. (2012). Primary care nursing role and care coordination: An observational study of nursing work in a community health center. *Online Journal of Issues in Nursing*, *17*(2).

26. Qian, S.-Y., Yu, P., Zhang, Z.-Y., Hailey, D. M., Davy, P. J., & Nelson, M. I. (2012). The work pattern of personal care workers in two Australian nursing homes: A time-motion study. *BMC Health Services Research*, *12*(1).

27. Gimeno, D., Barrientos-Gutiãrrez, T., Burau, K. D., & Felknor, S. A. (2012). Safety climate and verbal abuse among public hospital-based workers in Costa Rica. *Work*, *42*(1), 29–38.

28. Hsiao, J.-L., & Chen, R.-F. (2012). An investigation on task-technology fit of mobile nursing information systems for nursing performance. *CIN - Computers Informatics Nursing*, *30*(5), 265–273.

29. Beckman, T. J., Reed, D. A., Shanafelt, T. D., & West, C. P. (2012). Resident physician well-being and assessments of their knowledge and clinical performance. *Journal of General Internal Medicine*, *27*(3), 325–330.

30. Abbey, M., Chaboyer, W., & Mitchell, M. (2012). Understanding the work of intensive care nurses: A time and motion study. *Australian Critical Care*, *25*(1), 13–22.

31. Westbrook, J. I., Duffield, C., Li, L., & Creswick, N. J. (2011). How much time do nurses have for patients? A longitudinal study quantifying hospital nurses’ patterns of task time distribution and interactions with health professionals. *BMC Health Services Research*, *11*.

32. Greenslade, J. H., & Jimmieson, N. L. (2011). Organizational factors impacting on patient satisfaction: A cross sectional examination of service climate and linkages to nurses’ effort and performance. *International Journal of Nursing Studies*, *48*(10), 1188–1198.

33. Barker, L. M., & Nussbaum, M. A. (2011). The effects of fatigue on performance in simulated nursing work. *Ergonomics*, *54*(9), 815–829.

34. Yousefi, V. (2011). How Canadian hospitalists spend their time - A work-sampling study within a hospital medicine program in Ontario. *Journal of Clinical Outcomes Management*, *18*(4), 159–164.

35. Gardner, G., Gardner, A., Middleton, S., Della, P., Kain, V., & Doubrovsky, A. (2010). The work of nurse practitioners. *Journal of Advanced Nursing*, *66*(10), 2160–2169.

36. Williams, H., Harris, R., & Turner-Stokes, L. (2009). Work sampling: A quantitative analysis of nursing activity in a neuro-rehabilitation setting. *Journal of Advanced Nursing*, *65*(10), 2097–2107.

37. Westbrook, J. I., Ampt, A., Kearney, L., & Rob, M. I. (2008). All in a day’s work: An observational study to quantify how and with whom doctors on hospital wards spend their time. *Medical Journal of Australia*, *188*(9), 506–509.

38. Paquay, L., De Lepeleire, J., Milisen, K., Ylieff, M., Fontaine, O., & Buntinx, F. (2007). Tasks performance by registered nurses and care assistants in nursing homes: A quantitative comparison of survey data. *International Journal of Nursing Studies*, *44*(8), 1459–1467.

39. Greenslade, J. H., & Jimmieson, N. L. (2007). Distinguishing between task and contextual performance for nurses: Development of a job performance scale. *Journal of Advanced Nursing*, *58*(6), 602–611.

40. Fawcett, J., Schutt, R. K., Gail, G. B., Cruz, E. R., & Woodford, M. L. (2007). The work of nurse case managers in a Cancer and Cardiovascular Disease Risk Screening Program. *Professional Case Management*, *12*(2), 93–105.

41. Velasquez, D. (2007). The development and testing of a questionnaire to measure complexity of nursing work performed in nursing homes: NCCQ-NH. *Geriatric Nursing*, *28*(2), 90–98.

42. Roud, D., Giddings, L. S., & Koziol-McLain, J. (2005). A longitudinal survey of nurses’ self-reported performance during an entry to practice programme. *Nursing Praxis in New Zealand Inc.*, *21*(2), 37–46.

43. Van Der Weide, M., & Smits, J. (2004). Adoption of innovations by specialised nurses: Personal, work and organisational characteristics. *Health Policy*, *68*(1), 81–92.

44. Ogata, Y., Kobayashi, Y., Fukuda, T., Mori, K., Hashimoto, M., & Otosaka, K. (2004). Measuring relative work values for home care nursing services in Japan. *Nursing Research*, *53*(3), 145–153.

45. Skilbeck, J., & Seymour, J. (2002). Meeting complex needs: an analysis of Macmillan nurses’ work with patients. *International Journal of Palliative Nursing*, *8*(12), 574–582.

46. Hall, L. M., & O’Brien-Pallas, L. (2000). Redesigning nursing work in long-term care environments. *Nursing Economics*, *18*(2), 79–87.

47. Rasmussen, B. H., & Sandman, P. O. (2000). Nurses’ work in a hospice and in an oncological unit in Sweden. *The Hospice Journal*, *15*(1), 53–75.

48. Kitai, E., Kushnir, T., Herz, M., Melamed, S., Vigiser, D., & Granek, M. (1999). Correlation off work structure and job satisfaction among israeli family physicians. *Israel Medical Association Journal*, *1*(4), 236–240.

49. Upenieks, V. V. (1998). Work sampling. Assessing nursing efficiency. *Nursing Management*, *29*(4), 27–29.

50. Kushnir, T., Melamed, S., & Ribak, J. (1997). Occupational physicians in Israel: Work structure, job and personal characteristics, and job satisfaction. *Journal of Occupational and Environmental Medicine*, *39*(9), 874–881.

51. Gagnon, A. J., & Waghorn, K. (1996). Supportive Care by Maternity Nurses: A Work Sampling Study in an Intrapartum Unit. *Birth*, *23*(1), 1–6.

52. Giorgi, F., Mattei, A., Notarnicola, I., Petrucci, C., & Lancia, L. (2018). Can sleep quality and burnout affect the job performance of shift-work nurses? A hospital cross-sectional study. *Journal of Advanced Nursing*, *74*(3), 698–708.

53. Markon, M. P., Chiocchio, F., & Fleury, M. J. (2017). Modelling the effect of perceived interdependence among mental healthcare professionals on their work role performance. *Journal of Interprofessional Care*, *31*(4), 520–528.

54. Irwin, A., & Weidmann, A. E. (2015). A mixed methods investigation into the use of non-technical skills by community and hospital pharmacists. *Research in Social & Administrative Pharmacy: RSAP*, *11*(5), 675–685.

55. Binnewies, C., Sonnentag, S., & Mojza, E. J. (2009). Feeling recovered and thinking about the good sides of one’s work. *Journal of Occupational Health Psychology*, *14*(3), 243–256.

56. Pulich, M., & Tourigny, L. (2004). Workplace deviance: strategies for modifying employee behavior. *The Health Care Manager*, *23*(4), 290–301.

57. Bemus, A. M., Lindley, C. M., Sawyer, W. T., & McAllister III, J. C. (1996). Task analysis of home care pharmacy. *American journal of health-system pharmacy*, 53(23), 2831–2839.

58. Schweikhart, S. B. (1996). Reengineering the work of caregivers: role redefinition, team structures, and organizational redesign. *Hospital & Health Services Administration*, *41*(1), 19–36.

59. Bugaj, T. J., Nikendei, C., Groener, J. B., Stiepak, J., Huber, J., Moeltner, A., … Koechel, A. (2018). Ready to run the wards? - A descriptive follow-up study assessing future doctors’ clinical skills. *BMC Medical Education*, *18*(257), 1–8.

60. Seren, A. K. H., Tuna, R., & Bacaksiz, F. E. (2018) Reliability and Validity of the Turkish Version of the Job Performance Scale Instrument. *Journal of Nursing Research,26*(1), 27–35.

61. Rowan, B. H., Robinson, J., Granato, A., Bla, C. K., Kouyate, S., Djety, G. V., … Gloyd, S. (2018). Workforce patterns in the prevention of mother to child transmission of HIV in Cote d’Ivoire: a qualitative model. *Human Resources Health,16*(4).

62. Becton, J. B., Carr, J. C., Mossholder, K. W., & Walker, H. J. (2017). Differential Effects of Task Performance, Organizational Citizenship Behavior, and Job Complexity on Voluntary Turnover. *Journal of Business and Psychology*, *3* (4), 495–508.

63. Riskin, A., Erez, A., Foulk, T. A., Riskin-Geuz, K. S., Ziv, A., Sela, R., … Bamberger, P. A. (2017). Rudeness and Medical Team Performance. *Pediatrics*, *139*(2).

64. De Carlo, N. A., Dal Corso, L., Falco, A., Girardi, D., & Piccirelli, A. (2016). ``To be, rather than to seem”: The impact of supervisor’s and personal responsibility on work engagement, job performance, and job satisfaction in a positive healthcare organization. *TPM:Testing, Psychometrics, Methodology in Applied Psychology*, *23*(4), 561–580.

65. Chiocchio, F., Lebel, P., & Dube, J.-N. (2016). Informational role self-efficacy: a validation in interprofessional collaboration contexts involving healthcare service and project teams. *BMC Health Services Research*, *16*(1) 153.

66. Raglan, G. B., Margolis, B., Paulus, R. A., & Schulkin, J. (2015). Obstetrician/Gynecologists’ Experiences with Electronic Health Record Systems: A Narrative Study. *Journal of Reproductive Medicine*, *60*(3–4), 95–102.

67. Gordon, H. J., Demerouti, E., Bipp, T., & Le Blanc, P. M. (2015). The Job Demands and Resources Decision Making (JD-R-DM) Model. *European Journal of Work and Organizational Psychology*, *24*(1), 44–58.

68. Gordon, H. J., Demerouti, E., Le Blanc, P. M., Bakker, A. B., Bipp, T., & Verhagen, M. A. M. T. (2018). Individual job redesign: Job crafting interventions in healthcare. *Journal of Vocational Behavior*, *104*, 98–114.

69. Yousaf, A., Yang, H., & Sanders, K. (2015). Effects of intrinsic and extrinsic motivation on task and contextual performance of Pakistani professionals. *Journal of Managerial Psychology*, *30*(2), 133–150.

70. Calhoun, A. W., Boone, M. C., Dauer, A. K., Campbell, D. R., & Montgomery, V. L. (2014). Using simulation to investigate the impact of hours worked on task performance in an intensive care unit. *American Journal of Critical Care*, *23*(5), 387–395.

71. Chui, M. A., Look, K. A., & Mott, D. A. (2014). The association of subjective workload dimensions on quality of care and pharmacist quality of work life. *Research in Social & Administrative Pharmacy*, *10*(2), 328–340.

72. Feys, M., Anseel, F., & Wille, B. (2013). Responses to co-workers receiving recognition at work. *Journal of Managerial Psychology, 28*(5), 492–510.

73. Pohl, S., Dal Santo, L., & Battistelli, A. (2012). Perceived organizational support, job characteristics and intrinsic motivation as antecedents of organizational citizenship behaviours of nurses. *Revue internationale de psychologie sociale*, *25*(3), 39–52.

74. Flowerdew, L., Brown, R., Vincent, C., & Woloshynowych, M. (2012). Development and Validation of a Tool to Assess Emergency Physicians’ Nontechnical Skills. *ANNALS.*

75. Muse, L. A., & Wadsworth, L. L. (2012). An examination of traditional versus non-traditional benefits. *Journal of Managerial Psychology, 27*(2), 112–131.

76. Viitanen, J., Hypponen, H., Laaveri, T., Vanska, J., Reponen, J., & Winblad, I. (2011). National questionnaire study on clinical ICT systems proofs: Physicians suffer from poor usability. *International Journal of Medical Informatics*, *80*(10), 708–725.

77. Haun, S., Steinmetz, H., & Dormann, C. (2011). Objective work-nonwork conflict: From incompatible demands to decreased work role performance. *Journal of Vocational Behavior*, *79*(2), 578–587.

78. Veloski, J., Boex, J. R., Grasberger, M. J., Evans, A., & Wolfson, D. W. (2006). Systematic review of the literature on assessment, feedback and physicians’ clinical performance*: BEME Guide No. 7. *Medical Teacher*, *28*(2), 117–128.

79. Christensen, T., Faxvaag, A., Laerum, H., & Grimsmo, A. (2009). Norwegians GPs’ use of electronic patient record systems. *International Journal of Medical Informatics, 78*(12), 808–814.

80. Weigl, M., Mueller, A., Zupanc, A., & Angerer, P. (2009). Participant observation of time allocation, direct patient contact and simultaneous activities in hospital physicians. *BMC Health Services Research*, *9* (1)110.

81. Johnson, J., Truxillo, D. M., Erdogan, B., Bauer, T. N., & Hammer, L. (2009). Perceptions of Overall Fairness: Are Effects on Job Performance Moderated by Leader-Member Exchange? *Human Performance, 22*(5), 432–449.

82. Caldwell, B. S. (2008). Knowledge sharing and expertise coordination of event response in organizations. *Applied Ergonomics*, *39*, 427–438.

83. Cao, C. G. L., Weinger, M. B., Slagle, J., Zhou, C., Ou, J., Gillin, S., … Mazzei, W. (2008). Differences in day and night shift clinical performance in anesthesiology. *Human Factors*, *50*(2), 276–290.

84. Rotundo, M. (2002). The relative importance of task, citizenship, and counterproductive performance to global ratings of job performance: a policy-capturing approach. *The Journal of Applied Psychology*, *87*(1), 66–80.

85. Tang, Z., Weavind, L., Mazabob, J., Thomas, E. J., Chu-Weininger, M. Y. L., & Johnson, T. R. (2007). Workflow in intensive care unit remote monitoring: A time-and-motion study. *Critical Care Medicine, 35*(9), 2057–2063.

86. Yule, S., Flin, R., Paterson-Brown, S., Maran, N., & Rowley, D. (2006). Development of a rating system for surgeons’ non-technical skills. *Medical Education, 40*(11), 1098–1104.

87. Chisholm, C. D., Collison, E. K., Nelson, D. R., & Cordell, W. H. (2000). Emergency department workplace interruptions: Are emergency physicians ``interrupt-driven” and ``multitasking”? *Academic Emergency Medicine, 7*(11), 1239–1243.

88. Westbrook, J. I., Li, L., Shah, S., Lehnbom, E. C., Prgomet, M., Schofield, B., ... & Sheikh, A. (2019). A cross-country time and motion study to measure the impact of electronic medication management systems on the work of hospital pharmacists in Australia and England. *International Journal of Medical Informatics*.

89. Chen, F. L., Chen, K. C., Chiou, S. Y., Chen, P. Y., Du, M. L., & Tung, T. H. (2019). The longitudinal study for the work-related factors to job performance among nurses in emergency department. *Medicine*, *98*(12), e14950.

90. Palenzuela, P., Delgado, N., & Rodríguez, J. A. (2019). Exploring the Relationship between Contextual Performance and Burnout in Healthcare Professionals. Journal of Work and Organizational Psychology, 35(2), 115–121.

91. Low, Y. M., Sambasivan, M., & Ho, J. A. (2019). Impact of abusive supervision on counterproductive work behaviors of nurses. Asia Pacific Journal of Human Resources.

92. Michie, S., & West, M. A. (2004). Managing people and performance: an evidence based framework applied to health service organizations. International journal of management reviews, 5(2), 91–111.

## Appendix C

Table 8

**Table 8:** Overview Journals of publications

BMC Health Services Research	4
Journal of Advanced Nursing	4
International Journal of Medical Informatics	3
Journal of Managerial Psychology	3
Computers, Informatics, Nursing	2
International Journal of Nursing studies	2
Journal of vocational behavior	2
Nursing research	2
Research in social & administrative pharmacy	2
Acadamic emergency medicine	1
American Association of Critical-Care Nurses	1
American Journal of Health System Pharmacists	1
Annals of Emergency Medicine	1
Applied ergonomics	1
﻿Asia Pacific Journal of Human Resources	1
Asian Nurse research	1
Australian critical care	1
Birth	1
BMC Medical Education	1
BMJ Open	1
Critical Care medicine	1
Ergonomics	1
European Journal of Pediatrics	1
European Journal of Work and Organizational Psychology	1
Geriatric nursing	1
Health policy	1
Hospital & Health Service Administration	1
Human Factors	1
Human performance	1
Human Resources for Health	1
International Journal Health Planning Management	1
International Journal of Africa Nursing sciences	1
International Journal of Healthcare Quality Assurance	1
International Journal of Human Rights in healthcare	1
International Journal of Management Reviews	1
international Journal of Nursing Practice	1
International journal of Nursing sciences	1
International journal of palliative nursing	1
JCOMjournal	1
JGIM Journal of General Internal Medicine	1
Journal Nursing Care Quality	1
Journal of Business Psychology	1
Journal of clinical nursing	1
Journal of educational evaluation for health professionals	1
Journal of Hospital Medicine	1
journal of interprofessional care	1
Journal of Nursing Management	1
Journal of Occupational & Environmental Medicine	1
Journal of Occupational and Organizational Psychology	1
Journal of Occupational Health Psychology	1
Journal of Patient Safety	1
Journal of personnel psychology	1
Journal of Reproducive medicine	1
﻿Journal of Work and Organizational Psychology	1
Medical Education	1
Medicine	1
North American journal of medical sciences	1
Nursing economics	1
Nursing forum	1
Nursing management	1
Nursing Praxis in New Zealand	1
Pediatrics	1
Personnel review	1
Plos|one	1
Professional case Management	1
Research methodology	1
Revue internationale de psychologie sociale	1
The health Care Manager	1
The hospice journal	1
The Israel Medical Association Journal	1
The journal of applied business research	1
The Journal of Nursing research	1
The medical Journal of Australia	1
The online journal of issues in Nursing	1
TPM	1
Work	1
**Total**	**91**

## Abbreviations

CBA: Controlled Before After
cRCT: Cluster-Randomised Controlled Trial
ICROMS: Integrated quality Criteria for the Review Of Multiple Study designs
NCBA: Not Controlled Before After
NCITS: Non-Cotrolled Interrupted Time Series
OECD: Organisation for Economic Cooperation and Development
OSF: Open Science Framework
PRISMA: Preferred Reporting Items for Systematic Review and Meta-Analyses
RCT: Randomised Controlled Trial
